# Failure of the PTEN/aPKC/Lgl Axis Primes Formation of Adult Brain Tumours in* Drosophila*

**DOI:** 10.1155/2017/2690187

**Published:** 2017-12-27

**Authors:** Simona Paglia, Manuela Sollazzo, Simone Di Giacomo, Dario de Biase, Annalisa Pession, Daniela Grifoni

**Affiliations:** Department of “Pharmacy and Biotechnology”, University of Bologna, Via Selmi 3, 40126 Bologna, Italy

## Abstract

Different regions in the mammalian adult brain contain immature precursors, reinforcing the concept that brain cancers, such as glioblastoma multiforme (GBM), may originate from cells endowed with stem-like properties. Alterations of the tumour suppressor gene* PTEN* are very common in primary GBMs. Very recently,* PTEN* loss was shown to undermine a specific molecular axis, whose failure is associated with the maintenance of the GBM stem cells in mammals. This axis is composed of PTEN, aPKC, and the polarity determinant Lethal giant larvae (Lgl):* PTEN* loss promotes aPKC activation through the PI3K pathway, which in turn leads to Lgl inhibition, ultimately preventing stem cell differentiation. To find the neural precursors responding to perturbations of this molecular axis, we targeted different neurogenic regions of the* Drosophila* brain. Here we show that* PTEN* mutation impacts aPKC and Lgl protein levels also in* Drosophila*. Moreover, we demonstrate that PI3K activation is not sufficient to trigger tumourigenesis, while aPKC promotes hyperplastic growth of the neuroepithelium and a noticeable expansion of the type II neuroblasts. Finally, we show that these neuroblasts form invasive tumours that persist and keep growing in the adult, leading the affected animals to untimely death, thus displaying frankly malignant behaviours.

## 1. Introduction

Glioblastoma multiforme (GBM) is a highly malignant brain cancer whose prognosis is extremely poor [1]. As with other tumours [2], a subset of undifferentiated cells has been identified in GBM as “tumour-initiating cells” [3], due to their ability to originate a neoplastic mass resembling that of the donor patient when implanted in the brain of immunocompromised mice [4]. Primary GBMs represent about 95% of the total cases and develop as rapidly growing tumours with no evidence of premalignant lesions [5]. Several genetic alterations are recurrently implicated in primary GBM, among which* PTEN* inactivation is the most frequent, shown to occur through different mechanisms [6, 7].* PTEN* loss of function (LOF) promotes an increased PI3K signalling [8, 9] which activates, among others, the atypical Protein Kinase C (aPKC) [10, 11], resulting in alterations in tissue morphology both in* Drosophila* [12] and in mammalian cells [13]. Lethal giant larvae (Lgl), an aPKC substrate [14, 15], was first identified in* Drosophila* as an oncosuppressor protein [16] found at the membrane [17], encoded by the* lgl* gene, whose loss of function causes malignant growth of larval brain and epithelia [18, 19]. Of note, Lgl controls neuroblast (NBs) differentiation by regulating the asymmetric localisation of cell fate determinants in the neural progenitors [20–22] and phosphorylation by aPKC converts Lgl into an inactive form released in the cytoplasm [23].* Drosophila* Lgl is evolutionarily conserved [24], and our and other studies described its altered expression/localisation in several forms of human cancer [24–27]. Mammalian Lgl (Lgl1) is highly expressed in the brain and its knock-out causes severe brain dysplasia in mice [28]. Activated aPKC promotes GBM cell motility by dissociating Lgl1 from nonmuscle myosin II [29], and two recent papers demonstrated that inactivation of Lgl1 following* PTEN* loss promotes the maintenance of GBM stem cells in mammals [30, 31]. Consistently, Hugl1 (Human l*gl1*) overexpression in human GBM cells hampers their ability to form brain tumours in nude mice [32]. In the last fifteen years,* Drosophila* has been successfully used to investigate the genetic and molecular basis of different cancer hallmarks [33–35]. With regard to brain cancer, a model of glioma has been proposed that recapitulates some features of mammalian brain tumours [36, 37]. In this model, cancer was induced by activating the EGFR/PDGFR and PI3K pathways in glial cells, and the authors found that some neural cell types were not prone to neoplastic transformation [36], highlighting the relevance of the cell of origin to cancer initiation and progression [38]. A recent study demonstrated that the same molecular alterations give rise to different GBM subtypes when induced in different neural progenitors, pointing to the cell of origin as a major determinant of GBM diversity [39]. Moreover, GBM cell of origin was also shown to influence malignancy and drug sensitivity [40]. The* Drosophila* larval brain lobe contains several stem populations: the neuroepithelial (NE) cells, that originate the NBs of the optic lobe (OL) [41] by the same developmental mode as the mammalian forebrain [42], types I and II and mushroom bodies NBs of the central brain (CB) [43], and the newly reported non-NB progenitors that give rise to lamina glia and neurons [44]. Larval NBs undergo a limited number of asymmetric divisions and stop dividing before adulthood [45]; nevertheless, adult neurogenesis was recently observed in the medulla region of the OL, which increases following brain damage [46].* Drosophila* NBs have been extensively used as a model for brain cancer [47–49], and different genetic alterations were shown to prime specific NE/NB populations for tumourigenesis [50–55]. Among these cell populations, type II NBs are particularly attractive as their lineage is analogous to that of the mammalian neural stem cells, involving transient amplifying cells called Intermediate Neural Progenitors (INPs), used to expand the progenitor cell population [56, 57] and programmed cell death that culls excess neurons [58, 59]. Of note, type II NBs are known to undergo unrestrained growth [52, 60–63] and their lineages generate a variety of neurons and glial cells that contribute to the CB and the OL of the adult brain [64, 65]. Moreover, larval brains from* lgl* mutants produce primarily ectopic type II NBs [52, 55], prompting us to investigate the susceptibility of these stem cells to alterations of the PTEN/aPKC/Lgl axis. We first confirmed that* PTEN* LOF is sufficient to increase aPKC cortical loading and to inhibit Lgl membrane localisation in the* Drosophila* larval brain. We then manipulated the PTEN/aPKC/Lgl axis in the NE and in the type II NBs of the* Drosophila* brain, demonstrating that, while perturbation of this molecular pathway provokes mild NE hyperplasia, it triggers an accumulation of immature precursors in the larval central brain, where type II neuroblasts reside. In addition, these immature progenitors form adult brain tumours that kill the animals in time, thus showing a malignant behaviour.

## 2. Materials and Methods

### 2.1. Fly Stocks and Treatments

**Figure 1:**
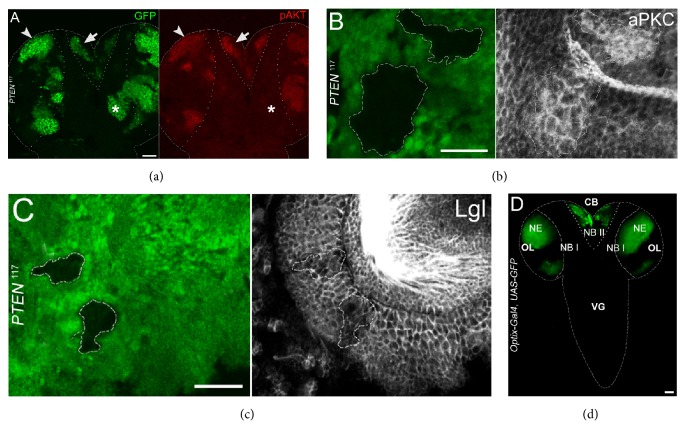
*PTEN* mutation activates the PI3K-pAKT pathway in different regions of the larval brain, while increasing aPKC and lowering Lgl at the cell membrane. (a) MARCM* PTEN*^*117*^ LOF clones (GFP^+^) induced in a wild-type background. pAKT staining (red) is positive in the optic lobes (outlined, arrowheads) and in the dorsomedial (DM) region of the brain (arrows). The asterisk marks a* PTEN*^*117*^ mutant clone in the central brain which does not activate AKT. ((b)-(c)) Flp/FRT* PTEN*^*117*^ clones in the OL show aPKC accumulation ((b) white, outlined) and Lgl decrease ((c) white, outlined). (d) Expression pattern of Optix in the larval brain: OL = optic lobe; CB = central brain; NE = neuroepithelium; NB I = type I NBs; NB II = type II NBs; VG = ventral ganglion. Scale bars are 50 *μ*m.

The following fly stocks were used in the study:* yw, hs-Flp, UAS-GFP, tub-Gal4; tub-Gal80, FRT40A-w; Ubi-GFPnls, FRT40A-w; PTEN*^*117*^*, FRT40A/CyO-yw, UAS-PI3K*^*CAAX*^*-w; UAS-*aPKC^CAAX-wt^*-w; Optix-Gal4, UAS-EGFP-w; Optix-Gal4, yvsc, UAS-mCD8::GFP; UAS-dmRNAi*. Fly lines were from the Bloomington Stock Center (NIH P40OD018537) except for* w*;* Optix-Gal4* II (A.H. Brand)-*w; PTEN*^*117*^*, FRT40A/CyO* (H. Stocker)-*w; *and* UAS-*aPKC^CAAX-wt^ (C.Q. Doe). Stocks and experimental crosses were all raised on standard medium at the temperatures indicated. Eggs were collected from 15 females in 8-hour time windows to avoid developmental delays due to overcrowding. For MARCM experiments (Figure 1(a)), where mutant cells are marked by GFP expression [66], larvae were heat-shocked for 20 minutes in a water bath at 37°C at 48 hours development, and for Flp/FRT experiments (Figures 1(b) and 1(c)), where mutant cells are marked by lack of GFP expression [67], larvae were heat-shocked for 60 minutes in a stove at 37°C at 48 hours development.

### 2.2. Immunohistochemistry

Larval and adult brains were dissected in PBS, fixed in 3.7% formaldehyde in PBS for 30 minutes, permeabilised in 0.5% Triton in PBS for 2 hours, and stained following standard protocols. Final samples were mounted in Fluoromount-G (Southern Biotechnology Associates, Inc.). The following primary antibodies were used: rabbit anti-aPKC*ζ* (1 : 200, sc-216, Santa Cruz Biotechnology); rabbit anti-Yki (1 : 400, K.D. Irvine); rabbit anti-phosphoAKT (1 : 200, Ser505, Cell Signaling Technology); rabbit anti-Lgl (1 : 500, D. Strand); rabbit anti-PntP1 (1 : 500, J.B. Skeath); rabbit anti-Mira (1 : 200, C.Q. Doe); rabbit anti-PH3 (1 : 200, Ser10 Upstate Biotechnology); mouse anti-MYC (1 : 5, P. Bellosta); mouse anti-dIAP1 (1 : 200, B.A. Hay); and mouse anti-Repo, anti-Elav, and anti-*γ*-H2AX (1 : 50, DSHB). Secondary antibodies were Alexa Fluor 555 goat anti-mouse and anti-rabbit (Invitrogen Corporation) and DyLight 649 goat anti-mouse and anti-rabbit (Jackson ImmunoResearch Laboratories).

### 2.3. Image and Statistical Analysis

Fluorescent images were taken on a Leica TCS SP2 confocal microscope, and the entire images were processed with Adobe Photoshop software; all the images shown are from a single *z* stack. ImageJ free software from NIH, Bethesda, MD, USA, was used to measure sample diameter and area. For statistical analysis, the number of samples is indicated in the figures. For IF analysis, the figures represent the average phenotype across 15–25 samples analysed, if not otherwise specified. Data represent mean ± s.d. Two-tailed Student's *t*-tests were used to determine significance. ^*∗∗*^*P* < 0.01. Graphs were created in GraphPad Prism 5.

## 3. Results and Discussion

### 3.1. PTEN Mutation Affects aPKC Abundance and Lgl Localisation in the* Drosophila* Brain

The phosphatase PTEN is known to regulate cell proliferation and growth through the PI3K/AKT pathway [8, 9, 68]. In cancer, deregulation of this signalling network supports a number of cellular characteristics such as survival, migration, and inability to differentiate [69]. This is partly achieved through activation of aPKC [10, 11], known to control cell polarity and asymmetric cell division in a variety of cells, from* Drosophila* to mammals [14, 19]. aPKC expression and activity are increased in human GBM [70], and its direct substrate Lgl has been recently associated with the maintenance of the GBM stem population [30, 31]. In* Drosophila*, PTEN is known to colocalise with the PAR/aPKC complex at the apical cortex of different cell types, where it serves multiple critical functions by helping maintain the correct actin organisation [12, 71]. With the aim to associate* PTEN* loss with Lgl inhibition in the* Drosophila* larval brain, we first investigated the impact of* PTEN* loss of function on the PI3K/AKT pathway in this organ. As can be seen in Figure 1(a), while the* PTEN* mutant clone (GFP^+^) in the ventrolateral (VL) CB failed to activate AKT (asterisk), clones within the OL (arrowhead) and in the dorsomedial (DM) CB (arrow) were positive to pAKT staining (red). These two regions of the larval brain contain the NE cells with their descendants (OL) and type II NBs (DM-CB), respectively. We then focused on the OL surface, where the NE cells and their progeny form a cohesive tissue and analysed aPKC and Lgl abundance and localisation in* PTEN* mutant clones. In Figure 1(b),* PTEN* mutant cells (GFP^−^, outlined) displayed aPKC membrane enrichment (outlined, compared with the surrounding cells). Lgl abundance was coherently lowered within the* PTEN* mutant clones in Figure 1(c) (GFP^−^, outlined). This was clear evidence that the PTEN/aPKC/Lgl axis is conserved in the* Drosophila* brain.

In* Drosophila*, the maternal contribution of mRNAs and proteins to the developing embryo is known to prevent detection of mutant phenotypes; in particular,* lgl* embryos have sufficient maternally provided transcript to enable animals to survive to the midlarval stage, after which their brain and epithelia undergo tumourigenic growth [18]. Complete depletion of Lgl is indeed required to trigger tumourigenesis and, being it a very stable protein [72], the use of* lgl* mutations or knock-down constructs may not be suitable to induce complete loss of function. Consistently,* PTEN* mutation, though lowering Lgl cellular levels, failed to deplete it completely (Figure 1(c)). To circumvent this issue, we genocopied* PTEN* and* lgl* loss of function by overexpressing their antagonists PI3K and aPKC. Since* PTEN* mutation activates the PI3K/AKT pathway in the OL and in the DM CB (Figure 1(a)), we directed kinase expression through the Optix promoter, which is active in subterritories of these regions (Figure 1(d)) [55, 73].

### 3.2. The Activated Form of aPKC Induces Hyperplastic Growth of the Larval NE

**Figure 2:**
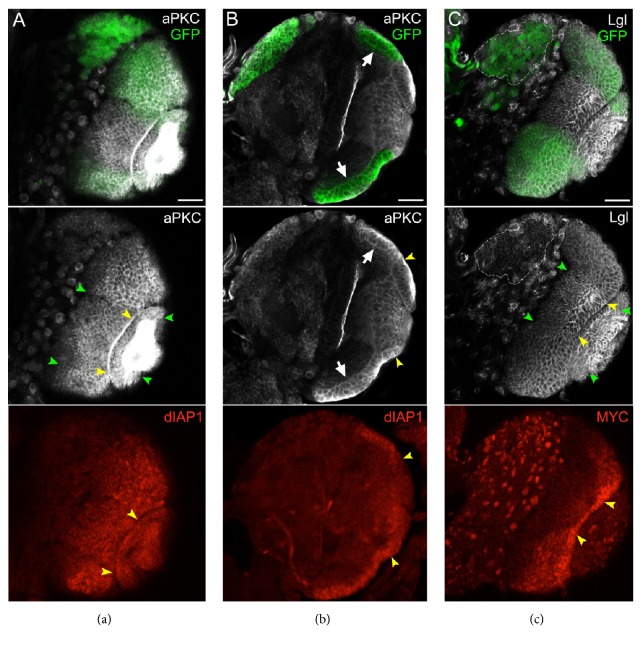
The activated form of aPKC induces hyperplastic growth of the larval NE. ((a)–(c)) Representative brains from Optix-aPKC^CAAX-wt^ late L3 larvae grown at 25°C. (a) Surface section showing aPKC (white) membrane enrichment (middle panel) and dIAP expression (lower panel) in the Optix NE domain (GFP^+^, upper panel). (b) Cross-section showing the multilayered structure of the NE and dIAP expression (lower panel) within the Optix domain (GFP^+^, upper panel). (c) Surface section displaying Lgl (white) release from the membrane (middle panel) and MYC expression (lower panel) within the Optix domain (GFP^+^). Green arrowheads indicate the boundaries between aPKC^CAAX-wt^ and wild-type OL, and yellow arrowheads indicate the boundaries between aPKC^CAAX-wt^ and wild-type NE. Scale bars are 50 *μ*m.

**Figure 3:**
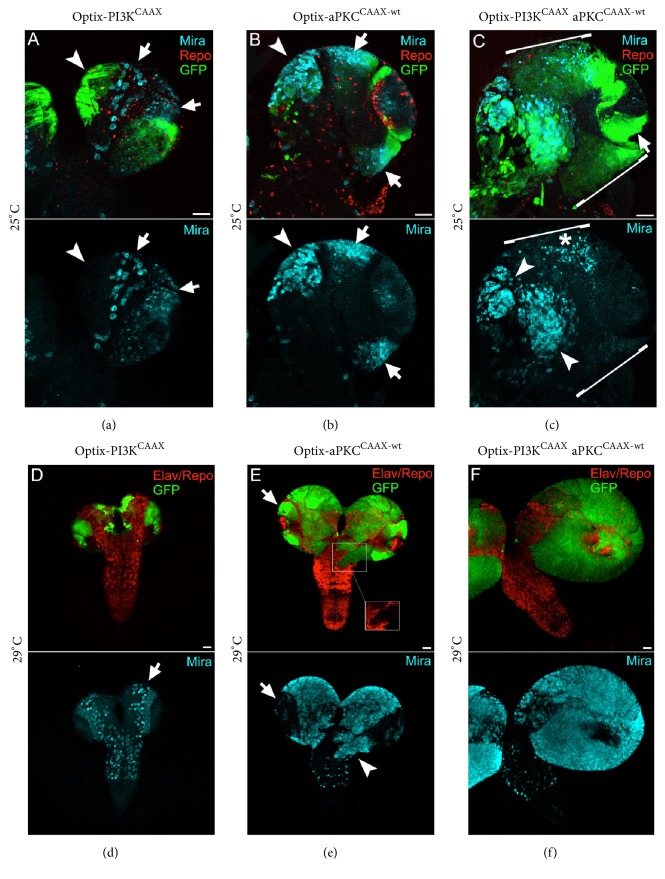
The activated form of aPKC induces neoplastic growth in type II NBs of the larval brain, and PI3K contributes to the overall growth. ((a)–(c)) Representative brains from Optix-PI3K (a), aPKC^CAAX-wt^ (b), and Optix-PI3K^CAAX^  aPKC^CAAX-wt^ (c) late L3 larvae grown at 25°C. Repo (red) stains glial cells and Mira (cyan) stains NBs. The lower panel shows Mira staining alone. While Mira marks mainly OL and CB type I NBs in (a) (arrows), aPKC activation triggers an increase in type II NBs (arrowhead) which form invasive masses (arrowheads) in cooperation with the active form of PI3K (c). ((d)–(f)) Representative brains from Optix-PI3K (d), aPKC^CAAX-wt^ (e), and Optix-  PI3K^CAAX^  aPKC^CAAX-wt^ (f) late L3 larvae grown at 29°C. Elav and Repo (red) stain neurons and glial cells, respectively, and Mira (cyan) stains NBs. The lower panel shows Mira staining alone. As it happens at 25°C, while Mira marks mainly OL and CB type I NBs in (a) (arrow), aPKC activation triggers a huge increase in type II NBs (arrowhead), which form invasive clusters (inset in the (e) upper panel) and grow as to fill the entire brain lobe in cooperation with the active form of PI3K (f). Scale bars are 50 *μ*m.

We first investigated the effect of aPKC activation in the OL. Expression of the aPKC^CAAX-wt^ transgene in the Optix domain provoked a substantial cellular increase of this kinase (Figure 2(a), compare the GFP^+^ and the GFP^−^ regions in the middle panel; the green and the yellow arrowheads mark the boundaries of the unaffected OL and NE, resp.). This confirmed that although Optix promoter is more active in the NE than in the OL NBs [73], it is however efficient in driving transgene expression also in these cells. Following aPKC activation, larval OL appeared normal, but the NE acquired a multilayered structure formed by rounded cells, as can be seen in Figure 2(b) (white arrows), where the cross-section of a brain hemisphere is shown. This phenotype was exacerbated by combined activation of PI3K and aPKC (Figure 3(c)) and was observed with variable severity in all the brains analysed (*n* = 23). In the middle panel, the yellow arrowheads mark the borders of the untargeted NE, which maintained the wild-type columnar shape (Figure 2(b)). In Figure 2(c), Lgl staining highlighted how this protein is released from the membrane following aPKC activation (compare the GFP^+^ and the GFP^−^ regions in the middle panel; the green and the yellow arrowheads mark the boundaries of the unaffected OL and NE, resp.), as it does in other* Drosophila* tissues [27]. Hyperplastic growth has also been reported in the Optix NE domain following Hippo pathway deregulation [73]. The Hippo pathway plays essential roles in regulating tissue growth [74] and is known to modulate proliferation and differentiation in the NE [75, 76]. Since aPKC and Lgl have been demonstrated to regulate growth through this signalling cascade in* Drosophila* [77, 78], it is conceivable that the hyperplastic phenotype triggered by aPKC activation in the NE is partly due to Hippo pathway deregulation: the downstream targets dIAP1 [79] and MYC [80, 81] showed indeed ectopic expression in the Optix OL domain (Figures 2(a), 2(b), and 2(c), lower panel; yellow arrowheads indicate the unaffected NE). Altogether, our findings indicate that, despite Lgl inhibition and Hippo pathway deregulation, the NE shows a mild morphological response, suggesting that its cells and their progeny, the OL NBs, are not prone to initiate brain cancer following alterations of the PTEN/aPKC/Lgl molecular axis.

### 3.3. The Activated Form of aPKC Primes Expansion of the Type II NBs of the Larval Brain, and PI3K Contributes to the Overall Organ Growth

We then shifted the focus from the NE to the other region that appeared to activate the PI3K/AKT pathway in response to* PTEN* mutation: type II NBs of the CB (see Figures 1(a) and 1(d)). This population is composed of 8 NBs/brain lobe, which represent in* Drosophila* the first identified postembryonic progenitors giving rise to both neurons and glial cells [64, 82]. Optix is expressed in 4 out of 8 of these NBs and respective progeny, in the DM region of the CB, and in 1 type I DM NB [55, 73]. We first analysed the proliferation versus differentiation phenotype of late L3 larvae grown at 25°C with single or combined activation of PI3K and aPKC in the Optix domain.  Figure 3(a) shows a larval brain lobe stained for Miranda (Mira), a NB marker, and reversed polarity (Repo), a pan-glial marker. Mira is an aPKC substrate which, such as Lgl, is released in the cytoplasm following phosphorylation; this in turn inhibits the correct segregation of polarity determinants and affects proper cell division [83]. Mira staining was mainly evident in the OL region and in type I NBs of the CB (arrows). Despite the promoter being active in the CB (GFP^+^, arrowhead), in these samples Mira staining was undetectable in type II NBs (Figure 3(a)), indicating that PI3K activation was not sufficient to induce their expansion. On the contrary, aPKC activation in the same regions drove a potent neurogenic wave in the CB (Figure 3(b), arrowhead) and an increase in Mira^+^ OL NBs number (arrows), promoting brain lobe overgrowth. Finally, activation of PI3K cooperated with aPKC in increasing both Mira^+^ tumour mass in the CB (Figure 3(c), lower panel, arrowheads) and overall brain size. Noticeably, the NE region and its progeny NBs underwent dramatic hyperplasia (arrow and brackets, GFP^+^, upper panel) but showed few signs of neurogenesis (asterisk, lower panel). We then repeated the same immunostainings as above on larval brains from crosses carried out at 29°C, to exacerbate cancer traits. Also in this case, Optix-PI3K^CAAX^ brains did not give origin to any aberrant phenotype, with OL and CB NBs found in stereotyped positions (Figure 3(d), arrow). Of note, aPKC expression at 29°C provoked a massive expansion of the CB NBs, which filled the entire brain lobes but the OLs, which were negative to Mira staining (Figure 3(e), arrow). This evidence confirmed that the expansion of the immature progenitors initiates from the CB NBs and does not involve the OL NBs. Another interesting trait observed in these samples was the formation of cell clusters invading the VG (arrowhead in the lower panel). These groups of invasive cells did not show any sign of differentiation, as they were negative to both Repo (glial marker) and Elav (neuronal marker) (Figure 3(e), upper panel, inset). This phenomenon was observed in 13 out of 18 Optix-aPKC^CAAX^ brains. Also at 29°C, activation of PI3K cooperated with aPKC in tumour development, with Mira^+^ cells filling the entire, oversized brain lobes (Figure 3(f), lower panel). In Supplementary Figure S1, a graph reports the average anterior-to-posterior (A/P) lobe diameter of the progeny from each experimental group. The differences between the three groups were all statistically significant, both at 25°C (Supplementary Figure S1A) and at 29°C (Supplementary Figure S1B). Finally, Supplementary Figure S2 represents Optix-aPKC^CAAX^ larval brains from crosses carried out at 29°C, where staining for PointedP1 (PntP1), a type II NBs marker [84, 85], revealed a large predominance of type II NBs and respective descendants in brain colonisation. In addition, these brain lobes presented a high degree of double strand breaks (Supplementary Figure S2), underlining defective DNA repair, typical of malignant tumours [86] and, interestingly, of GBM stem cells [87]. Altogether, these analyses performed on larval brains suggest a strong implication for aPKC in cancer initiation from type II NBs. Moreover, aPKC cortical activity in* lgl*^−/−^ INPs originated from type II NBs is known to revert these cells back into NBs [88], and here we showed that, also in wild-type brains, while aPKC activation does not seem to initiate tumour growth from the NE-derived NBs, it promotes a huge expansion of the type II NBs, which eventually colonise the entire CBs at 29°C.

### 3.4. The Unrestrained Growth Initiated in Type II NBs by the Activated Form of aPKC Leads to Formation of Adult Brain Tumours

**Figure 4:**
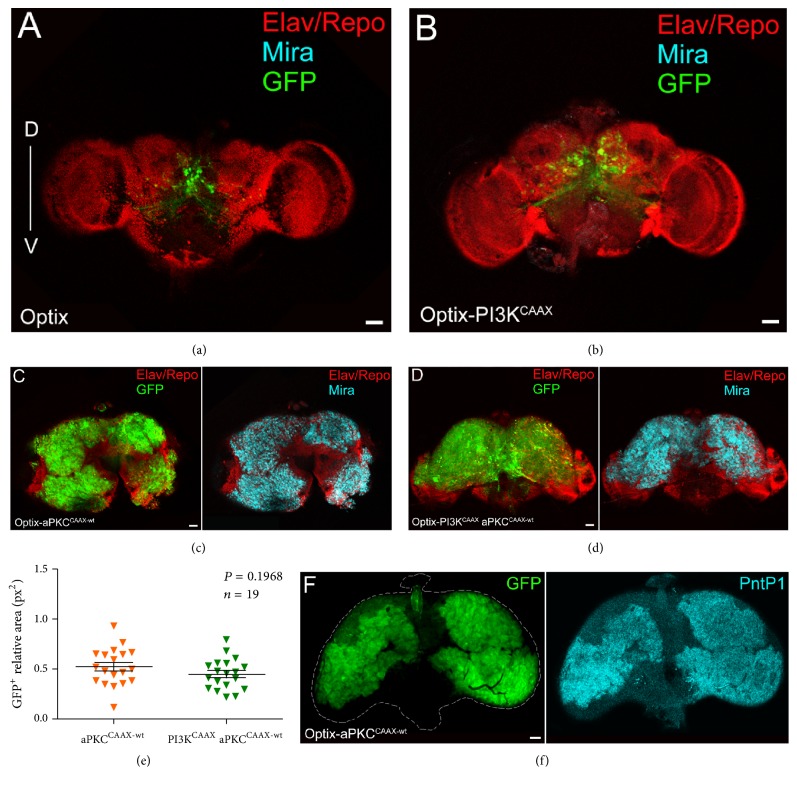
The neoplastic growth induced in type II NBs of the larval brain by the activated form of aPKC leads to formation of adult brain tumours. ((a)–(d)) Representative adult brains from Optix (a), Optix-PI3K (b), aPKC^CAAX-wt^ (c), and Optix-PI3K^CAAX^  aPKC^CAAX-wt^ (d) 1–4-day-old individuals. All the samples are dorsal up and ventral down. While the a-Mira antibody does not stain control (a) and Optix-PI3K^CAAX^ (b) brains, a myriad Mira-positive cells can be observed both in aPKC^CAAX-wt^ (c) and in Optix-PI3K^CAAX^  aPKC^CAAX-wt^ (d) samples (cyan). Elav and Repo staining is shown in red. (e) Graph displaying the ratio of the total GFP^+^ area normalised to the whole brain area for the two indicated groups. Each triangle represents one brain, and the central bar indicates the average ratio. The two sample groups are not statistically different, *P* = 0.1968. (f) Adult brains from Optix-aPKC^CAAX-wt^ 1–4-day-old individuals showing PntP1 staining. Scale bars are 50 *μ*m.

**Figure 5:**
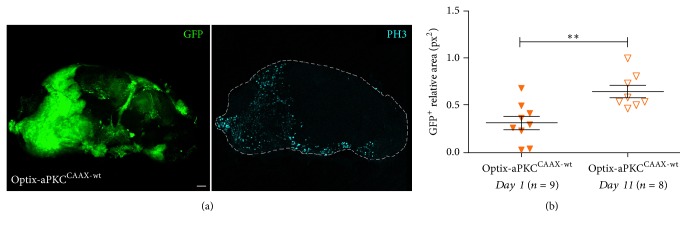
The brain tumours keep growing during the adult life. (a) Adult brains from Optix-aPKC^CAAX-wt^ 1–4-day-old individuals showing PH3 staining. The brain is outlined in (a), right image. Scale bar is 50 *μ*m. (b) Graph displaying the ratio of the total GFP^+^ area normalised to the whole brain area for the two indicated groups. Each triangle represents one brain, and the central bar indicates the average ratio. The two sample groups are statistically different, *P* < 0.05.

In* Drosophila*, adult brain tumours have been observed following inactivation of the translational repressor Brat, the transcription factor Earmuff, or proteins of the SWI chromatin complex in type II NBs and INPs [61, 89–92]. We thus observed adult flies carrying single or combined ectopic activation of PI3K and aPKC. As can be seen in Supplementary Figure S3A, the differences between the observed and expected progeny numbers were not significant at 25°C. It is however important to underline that 1/3 of the Optix-PI3K^CAAX^  aPKC^CAAX-wt^ progeny died as pharate adults; therefore the following analyses will possibly not include the most severe phenotypes of this class. At 29°C, no Optix-aPKC^CAAX-wt^ and Optix-PI3K^CAAX^  aPKC^CAAX-wt^ eclosed adults were recovered (Supplementary Figure S3A). We therefore proceeded by analysing all the progenies at 25°C. Optix-aPKC^CAAX-wt^ animals displayed small, cone-shaped eyes (Supplementary Figure S3C, middle panel), possibly due to kinase activation in the eye disc [93]; therefore we measured the head capsule width (IOD, Interocular Distance) for each class, normalised to that of control siblings, to find differences among the three samples. The graph in Supplementary Figure S3B indicates that Optix-aPKC^CAAX-wt^ fly heads were much larger than those of the other two classes, as can be appreciated in Supplementary Figure S3C. An analysis of these three classes of flies under a fluorescence stereoscope revealed that while Optix-PI3K^CAAX^ fly heads were negative, both Optix-aPKC^CAAX-wt^ and Optix-PI3K^CAAX^  aPKC^CAAX-wt^ fly heads showed the presence of GFP^+^ masses in the CB in about 30% of the scored individuals. Those individuals were found to display the highest IOD values within their class (not shown), suggesting this measure may be utilised as an index of brain tumour growth. We then analysed the phenotypes of adult brains from 1–4-day-old flies grown at 25°C with single or combined activation of PI3K and aPKC. As illustrated in Figure 4(a), residual Optix reporter activity was visible in some cells, and the same was observed in Optix-PI3K^CAAX^ adult brains (Figure 4(b)), where the Optix domain appeared slightly enlarged. The GFP^+^ cells were Mira^−^ in both samples, indicating that the adult brains did not contain detectable immature cells. On the contrary, both Optix-aPKC^CAAX-wt^ (Figure 4(c)) and Optix-PI3K^CAAX^  aPKC^CAAX-wt^ (Figure 4(d)) adult brains contained a myriad GFP^+^Mira^+^ cells, representing immature neural progenitors which failed to respond to cell cycle termination signals at the onset of metamorphosis, hence their persistence in the adult. A statistical analysis of the GFP^+^ areas in the Optix-aPKC^CAAX-wt^ versus the Optix-PI3K^CAAX^  aPKC^CAAX-wt^ samples did not reveal significant differences (Figure 4(e)), but we speculate that the combined activation of the two kinases did not allow the most compromised animals to eclose, escaping this analysis (see Supplementary Figure S3). A staining for PntP1 revealed that the GFP^+^ immature cells contained in the Optix-aPKC^CAAX-wt^ adult brains were type II NBs/INPs: an analysis performed on 9 Optix-aPKC^CAAX-wt^ adult brains indeed confirmed that all the GFP^+^ brain areas were also PntP1^+^. This was convincing evidence that aPKC cortical activation initiates tumourigenesis in type II lineages, as it is for other proteins involved in NB polarity determination [89, 90]. With the aim to understand if these brain tumours were mitotically active, we stained Optix-aPKC^CAAX-wt^ adult brains for the Phospho-Histone H3, an immunomarker specific for cells undergoing cell division. As can be seen in Figure 5(a), GFP-positive areas show diffuse PH3 staining, suggesting these tumours are still proliferating in the adult. A comparison of the average GFP^+^ area in brains from flies of different age indeed found that brain tumours from 11-day-old flies were 1.5-fold bigger than brain tumours from 1-day-old flies (Figure 5(b)), confirming these brain cancers keep growing during adult life. Finally, we calculated the average survival of Optix-aPKC^CAAX-wt^ adult flies over time and, at 30 days from eclosion, we found that, while the 83% of control siblings were alive and healthy, only the 20% of the experimental flies were alive, with clear signs of cancer burden such as scarce motility and inability to feed and mate (see Supplementary Figure S4 for survival curve). Altogether, these results demonstrate that aPKC cortical recruitment primes in the type II NB lineages a series of molecular events which promote the accumulation of immature progenitors in the larval CB. These undifferentiated masses continue to proliferate, escape proper controls during metamorphosis, and persist in the adult, where they keep growing and lead the animals to untimely death, thus behaving like frankly malignant tumours.

### 3.5. Brain Tumour Growth Induced by aPKC Activation Is MYC-Dependent

**Figure 6:**
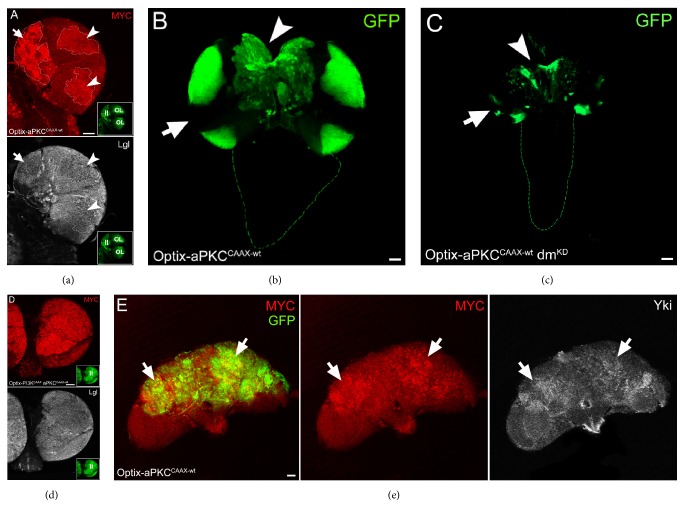
Brain tumour growth induced by aPKC activation is MYC-dependent. ((a)-(b)) Representative brains from Optix-aPKC^CAAX-wt^ larvae grown at 25°C. (a) MYC (red) and Lgl (white) staining. Regions II and OL in the GFP^+^ inset indicate type II and OL NBs, respectively. (b) The arrow indicates the NE-medulla region and the arrowhead points to the type II NBs in the DM region. (c) The same regions as in (b) are indicated in an Optix-aPKC^CAAX-wt^  dm^KD^ brain. (d) Representative brains from Optix-PI3K^CAAX^  aPKC^CAAX-wt^ larvae grown at 25°C stained for MYC (red) and Lgl (white). The GFP^+^ inset shows the huge expansion of the type II NBs (region II). (e) Optix-aPKC^CAAX-wt^ representative adult brains from 1–4-day-old animals showing MYC upregulation associated with Yki accumulation (arrows). Scale bars are 50 *μ*m.

Neural progenitor cells need MYC function to proliferate properly [94], and MYC family proteins are highly deregulated in human brain cancers, GBM included [95–97]. Brat promotes type II NBs asymmetric cell division by repressing MYC, and expression of the human orthologue TRIM3 represses c-MYC activity in GBM cells [98]. Direct MYC inactivation or inhibition of MYC-driven processes has been shown to impair GBM growth in several ways [99–101]. aPKC activation promotes MYC ectopic expression in the OL (Figure 2(c), lower panel); therefore we investigated MYC abundance in the larval CB following kinase activation.  Figure 6(a) represents an Optix-aPKC^CAAX-wt^ larval brain lobe where, in the regions showing lower Lgl levels (outlined, lower panel), MYC was aberrantly expressed. The highest MYC levels were visible in the CB (arrow), indicated as region II in the inset, but MYC was found deregulated, as above described, also in the OL NBs (Figure 6(a), arrowheads). MYC knockdown in Optix-aPKC^CAAX-wt^ individuals deeply impaired tumour development (compare Figures 6(c) and 6(b), arrows) and reverted the organ back to wild-type dimensions (compare Figure 6(c) with Figure 1(d)), demonstrating that these tumours depend on MYC for both initiation and progression. PI3K^CAAX^ cooperates with aPKC^CAAX-wt^ also in MYC deregulation, as can be appreciated in Figure 6(d), where the inset indicates the CB region as II.

Finally, we examined MYC levels in Optix-aPKC^CAAX-wt^ adult brains and we found it was ectopically expressed in several tumour areas (Figure 6(e), arrows). In the same areas, some cells overexpressed Yki, the downstream effector of the Hippo pathway [79], suggesting that aPKC-mediated tumourigenesis in type II NBs/INPs may be partly mediated by this pathway. In summary, these results indicate that deregulation of the Hippo pathway and of its target MYC contribute to the tumourigenic growth promoted by aPKC activation in type II NBs.* bantam*, another Hippo target [102], has also been found to control differentiation of both type I and type II NBs [63, 103]; thus reinforcing the evidence that the Hippo signalling cascade, by connecting polarity and growth regulators, may orchestrate different aspects of brain cancer development. The Yki human orthologue YAP is indeed found overexpressed in a number of human cancers [104], including GBM [105]. Moreover, it is known that aPKC activation increases MYC levels through deregulation of the Hippo pathway both in* Drosophila* [77] and in mammals [106], while PI3K activation is known to regulate MYC stability and MYC-dependent transcription in* Drosophila* [107, 108], as it happens in mammals [109]. Finally, c-MYC is known to inhibit* PTEN* by upregulating miR-26A in GBM [110], thus creating a vicious circle.

## 4. Conclusions

Primary glioblastoma (GBM) is the most common and incurable brain cancer of the adult, displaying high cellular and genetic diversity, used to define tumour subtypes [111]. GBM origin is long being debated, although the most likely hypothesis is that it may initiate from different cells, making it difficult to find a treatment for such a heterogeneous disease [1]. Given the presence of cancer stem cells in GBM, which reside in perivascular niches [112] and resist DNA-damaging therapies [87], interest is growing towards their specific biology. For this reason, investigations on the mechanisms reprogramming normal neural progenitors into cancer stem cells are fundamental to decipher the essential logic driving brain cancer development at the genetic, molecular, and cellular levels.* PTEN* deficiency has proven to be sufficient to reprogramme human neural progenitors into GBM stem cells [113] and its inactivation is very frequent in GBM, occurring through a number of different mechanisms [7, 110, 114]. We focused our attention on recent studies that associated alterations in the PTEN/aPKC/Lgl axis with the maintenance of GBM stem cells [30, 31]. This axis regulates cell growth and cell polarity, two essential features that guarantee proper differentiation of neural stem cells, through combined action of the conserved PI3K/AKT and aPKC/Lgl pathways [115].* Drosophila* is routinely used as a model for the study of cancer biology [33], so we investigated the consequences of alterations in these pathways on different neural progenitors found in the* Drosophila* brain. We first confirmed that* PTEN* deficiency in the fly brain is able to activate aPKC with a consistent inhibition of Lgl (Figures 1(b) and 1(c)). We then expressed an activated form of aPKC in the optic lobe and observed hyperplastic growth of the neuroepithelium that switched from the wild-type columnar monolayer into a multilayer of rounded cells, without evident morphological alterations of the brain lobe (Figure 2). Of note, neuroepithelium hyperplasia was associated with ectopic expression of the Hippo pathway downstream targets dIAP1 and MYC in the neuroblasts (NBs) of the optic lobe (Figure 2, lower panel), indicating that loss of cell polarity in the neuroepithelium affects differentiation [75, 76]. aPKC activation, alone or combined with PI3K, caused instead severe phenotypes in the central brain. In that region, type II NBs originate neurons and glial cells through transient amplifying cells, as it is for mammalian neural stem cells [56, 57]. While PI3K activation did not hamper NB differentiation, expression of aPKC alone or in combination with PI3K promoted a dramatic expansion of the neural progenitor cells, which eventually filled the central brain (Figure 3) and persisted in the adult (Figure 4), where they kept growing (Figure 5) leading the animal to premature death. The Hippo signalling cascade was found deregulated also in the adult brains (Figure 6(e)), suggesting an involvement of this central pathway in the integration of multiple signals during brain tumourigenesis. Our neurogenic model of brain cancer in the fly seems to recapitulate a number of traits typical of human brain cancers. Thanks to the use of more sophisticated genetic systems, it may help identify and characterise the neural lineage most susceptible to* PTEN* inactivation. Future work is therefore warranted to address the many open questions on the genesis and biology of GBM.

## Supplementary Materials

Supplementary Materials Supplementary Figure S1. Measurement of the A-P larval brain lobe diameter following neurogenic growth. Supplementary Figure S2. The most part of the immature cells filling the Optix-aPKC^CAAX-wt^ larval brains are type II NBs and INPs. Supplementary Figure S3. The activated form of aPKC induces an increase of the adult fly interocular distance (IOD). Supplementary Figure S4. Kaplan-Meier survival curve of Optix-aPKC^CAAX-wt^ adult flies maintained at 25°C.

## References

[1] Clarke J., Butowski N., Chang S. (2010). Recent advances in therapy for glioblastoma. *JAMA Neurology*.

[2] Sell S. (2010). On the stem cell origin of cancer. *The American Journal of Pathology*.

[3] Galli R., Binda E., Orfanelli U. (2004). Isolation and characterization of tumorigenic, stem-like neural precursors from human glioblastoma. *Cancer Research*.

[4] Muñoz D. M., Guha A. (2011). Mouse models to interrogate the implications of the differentiation status in the ontogeny of gliomas. *Oncotarget *.

[5] Ohgaki H., Dessen P., Jourde B. (2004). Genetic pathways to glioblastoma: a population-based study. *Cancer Research*.

[6] Verhaak R. G. W., Hoadley K. A., Purdom E. (2010). Integrated genomic analysis identifies clinically relevant subtypes of glioblastoma characterized by abnormalities in PDGFRA, IDH1, EGFR, and NF1. *Cancer Cell*.

[7] Zhang L., Zhang S., Yao J. (2015). Microenvironment-induced PTEN loss by exosomal microRNA primes brain metastasis outgrowth. *Nature*.

[8] Stambolic V., Suzuki A., de la Pompa J. L. (1998). Negative regulation of PKB/Akt-dependent cell survival by the tumor suppressor PTEN. *Cell*.

[9] Sun H., Lesche R., Li D.-M. (1999). PTEN modulates cell cycle progression and cell survival by regulating phosphatidylinositol 3,4,5,-trisphosphate and Akt/protein kinase B signaling pathway. *Proceedings of the National Acadamy of Sciences of the United States of America*.

[10] Nakanishi H., Brewer K. A., Exton J. H. (1993). Activation of the *ζ* isozyme of protein kinase C by phosphatidylinositol 3,4,5-trisphosphate. *The Journal of Biological Chemistry*.

[11] Le Good J. A., Ziegler W. H., Parekh D. B., Alessi D. R., Cohen P., Parker P. J. (1998). Protein kinase C isotypes controlled by phosphoinositide 3-kinase through the protein kinase PDK1. *Science*.

[12] von Stein W., Ramrath A., Grimm A., Müller-Borg M., Wodarz A. (2005). Direct association of Bazooka/PAR-3 with the lipid phosphatase PTEN reveals a link between the PAR/aPKC complex and phosphoinositide signaling. *Development*.

[13] Martin-Belmonte F., Gassama A., Datta A. (2007). PTEN-mediated apical segregation of phosphoinositides controls epithelial morphogenesis through Cdc42. *Cell*.

[14] Plant P. J., Fawcett J. P., Lin D. C. C. (2003). A polarity complex of mPar-6 and atypical PKC binds, phosphorylates and regulates mammalian Lgl. *Nature Cell Biology*.

[15] Betschinger J., Mechtler K., Knoblich J. A. (2003). The Par complex directs asymmetric cell division by phosphorylating the cytoskeletal protein Lgl. *Nature*.

[16] Gateff E. (1978). Malignant neoplasms of genetic origin in Drosophila melanogaster. *Science*.

[17] Klämbt C., Schmidt O. (1986). Developmental expression and tissue distribution of the lethal (2) giant larvae protein of Drosophila melanogaster. *EMBO Journal*.

[18] Bilder D. (2004). Epithelial polarity and proliferation control: links from the Drosophila neoplastictumor suppressors. *Genes and Development*.

[19] Rolls M. M., Albertson R., Shih H.-P., Lee C.-Y., Doe C. Q. (2003). Drosophila aPKC regulates cell polarity and cell proliferation in neuroblasts and epithelia. *The Journal of Cell Biology*.

[20] Wirtz-Peitz F., Knoblich J. A. (2006). Lethal giant larvae take on a life of their own. *Trends in Cell Biology*.

[21] Ohshiro T., Yagami T., Zhang C., Matsuzaki F. (2000). Role of cortical tumour-suppressor proteins in asymmetric division of *Drosophila* neuroblast. *Nature*.

[22] Peng C.-Y., Manning L., Albertson R., Doe C. Q. (2000). The tumour-suppressor genes lgl and dlg regulate basal protein targeting in Drosophila neuroblasts. *Nature*.

[23] Betschinger J., Eisenhaber F., Knoblich J. A. (2005). Phosphorylation-induced autoinhibition regulates the cytoskeletal protein Lethal (2) giant larvae. *Current Biology*.

[24] Grifoni D., Garoia F., Schimanski C. C. (2004). The human protein Hugl-1 substitutes for Drosophila lethal giant larvae tumour suppressor function in vivo. *Oncogene*.

[25] Kuphal S., Wallner S., Schimanski C. C. (2006). Expression of Hugl-1 is strongly reduced in malignant melanoma. *Oncogene*.

[26] Schimanski C. C., Schmitz G., Kashyap A. (2005). Reduced expression of Hugl-1, the human homologue of Drosophila tumour suppressor gene lgl, contributes to progression of colorectal cancer. *Oncogene*.

[27] Grifoni D., Garoia F., Bellosta P. (2007). aPKC*ζ* cortical loading is associated with Lgl cytoplasmic release and tumor growth in Drosophila and human epithelia. *Oncogene*.

[28] Klezovitch O., Fernandez T. E., Tapscott S. J., Vasioukhin V. (2004). Loss of cell polarity causes severe brain dysplasia in Lgl1 knockout mice. *Genes & Development*.

[29] Baldwin R. M., Barrett G. M., Parolin D. A. E. (2010). Coordination of glioblastoma cell motility by PKCiota. *Molecular Cancer*.

[30] Gont A., Hanson J. E. L., Lavictoire S. J. (2014). Inhibition of glioblastoma malignancy by Lgl1. *Oncotarget *.

[31] Gont A., Hanson J. E. L., Lavictoire S. J. (2013). PTEN loss represses glioblastoma tumor initiating cell differentiation via inactivation of Lgl1. *Oncotarget *.

[32] Liu X., Lu D., Ma P. (2015). Hugl-1 inhibits glioma cell growth in intracranial model. *Journal of Neuro-Oncology*.

[33] Gonzalez C. (2013). *Drosophila melanogaster*: a model and a tool to investigate malignancy and identify new therapeutics. *Nature Reviews Cancer*.

[34] Sonoshita M., Cagan R. L. (2017). Modeling human cancers in Drosophila. *Current Topics in Developmental Biology*.

[35] Hou S. X., Singh S. R. (2017). Stem-cell-based tumorigenesis in adult Drosophila. *Current Topics in Developmental Biology*.

[36] Read R. D., Cavenee W. K., Furnari F. B., Thomas J. B. (2009). A Drosophila model for EGFR-Ras and PI3K-dependent human glioma. *PLoS Genetics*.

[37] Witte H. T., Jeibmann A., Klämbt C., Paulus W. (2009). Modeling glioma growth and invasion in Drosophila melanogaster. *Neoplasia*.

[38] Chesler D. A., Berger M. S., Quinones-Hinojosa A. (2012). The potential origin of glioblastoma initiating cells. *Frontiers in Bioscience - Scholar*.

[39] Alcantara Llaguno S. R., Wang Z., Sun D. (2015). Adult lineage-restricted CNS progenitors specify distinct glioblastoma subtypes. *Cancer Cell*.

[40] Jiang Y., Marinescu V. D., Xie Y., Jarvius M., Maturi N. P., Haglund C. (2017). Glioblastoma cell malignancy and drug sensitivity are affected by the cell of origin. *Cell Reports*.

[41] Egger B., Boone J. Q., Stevens N. R., Brand A. H., Doe C. Q. (2007). Regulation of spindle orientation and neural stem cell fate in the Drosophila optic lobe. *Neural Development*.

[42] Götz M., Huttner W. B. (2005). The cell biology of neurogenesis. *Nature Reviews Molecular Cell Biology*.

[43] Homem C. C. F., Knoblich J. A. (2012). Drosophila neuroblasts: a model for stem cell biology. *Development*.

[44] Chen Z., Del Valle Rodriguez A., Li X., Erclik T., Fernandes V. M., Desplan C. (2016). A unique class of neural progenitors in the drosophila optic lobe generates both migrating neurons and glia. *Cell Reports*.

[45] Truman J. W., Bate M. (1988). Spatial and temporal patterns of neurogenesis in the central nervous system of Drosophila melanogaster. *Developmental Biology*.

[46] Fernández-Hernández I., Rhiner C., Moreno E. (2013). Adult neurogenesis in Drosophila. *Cell Reports*.

[47] Li S., Wang H., Groth C. (2014). Drosophila neuroblasts as a new model for the study of stem cell self-renewal and tumour formation. *Bioscience Reports*.

[48] Jiang Y., Reichert H. (2014). Drosophila neural stem cells in brain development and tumor formation. *Journal of Neurogenetics*.

[49] Saini N., Reichert H. (2012). Neural stem cells in drosophila: Molecular genetic mechanisms underlying normal neural proliferation and abnormal brain tumor formation. *Stem Cells International*.

[50] Richter C., Oktaba K., Steinmann J., Müller J., Knoblich J. A. (2011). The tumour suppressor L(3)mbt inhibits neuroepithelial proliferation and acts on insulator elements. *Nature Cell Biology*.

[51] Lee C.-Y., Robinson K. J., Doe C. Q. (2006). Lgl, Pins and aPKC regulate neuroblast self-renewal versus differentiation. *Nature*.

[52] Bowman S. K., Rolland V., Betschinger J., Kinsey K. A., Emery G., Knoblich J. A. (2008). The tumor suppressors brat and numb regulate transit-amplifying neuroblast lineages in drosophila. *Developmental Cell*.

[53] Komori H., Xiao Q., McCartney B. M., Lee C.-Y. (2014). Brain tumor specifies intermediate progenitor cell identity by attenuating *β*-catenin/Armadillo activity. *Development*.

[54] Rives-Quinto N., Franco M., de Torres-Jurado A., Carmena A. (2017). Canoe and scribble loss synergizes causing tumor-like overgrowth via ras activation in neural stem cells and epithelia. *Development*.

[55] Carney T. D., Miller M. R., Robinson K. J., Bayraktar O. A., Osterhout J. A., Doe C. Q. (2012). Functional genomics identifies neural stem cell sub-type expression profiles and genes regulating neuroblast homeostasis. *Developmental Biology*.

[56] Boone J. Q., Doe C. Q. (2008). Identification of Drosophila type II neuroblast lineages containing transit amplifying ganglion mother cells. *Developmental Neurobiology*.

[57] Ming G. L., Song H. (2011). Adult neurogenesis in the mammalian brain: significant answers and significant questions. *Neuron*.

[58] Jiang Y., Reichert H. (2012). Programmed cell death in type II neuroblast lineages is required for central complex development in the Drosophila brain. *Neural Development*.

[59] Ryu J. R., Hong C. J., Kim J. Y., Kim E.-K., Sun W., Yu S.-W. (2016). Control of adult neurogenesis by programmed cell death in the mammalian brain. *Molecular Brain*.

[60] Ouyang Y., Petritsch C., Wen H., Jan L., Jan Y. N., Lu B. (2011). Dronc caspase exerts a non-apoptotic function to restrain phospho-Numb-induced ectopic neuroblast formation in Drosophila. *Development*.

[61] Koe C. T., Li S., Rossi F. (2014). The Brm-HDAC3-Erm repressor complex suppresses dedifferentiation in Drosophila type II neuroblast lineages. *eLife*.

[62] Li X., Xie Y., Zhu S. (2016). Notch maintains Drosophila type II neuroblasts by suppressing expression of the fez transcription factor earmuff. *Development*.

[63] Wu Y., Lee K., Song Y., Gehrke S., Lu B., Moore A. W. (2017). The bantam microRNA acts through Numb to exert cell growth control and feedback regulation of Notch in tumor-forming stem cells in the Drosophila brain. *PLoS Genetics*.

[64] Viktorin G., Riebli N., Popkova A., Giangrande A., Reichert H. (2011). Multipotent neural stem cells generate glial cells of the central complex through transit amplifying intermediate progenitors in Drosophila brain development. *Developmental Biology*.

[65] Bayraktar O. A., Doe C. Q. (2013). Combinatorial temporal patterning in progenitors expands neural diversity. *Nature*.

[66] Lee T., Luo L. (2001). Mosaic analysis with a repressible cell marker (MARCM) for Drosophila neural development. *Trends in Neurosciences*.

[67] Xu T., Rubin G. M. (1993). Analysis of genetic mosaics in developing and adult Drosophila tissues. *Development*.

[68] Maehama T., Dixon J. E. (1998). The tumor suppressor, PTEN/MMAC1, dephosphorylates the lipid second messenger, phosphatidylinositol 3,4,5-trisphosphate. *The Journal of Biological Chemistry*.

[69] Mantamadiotis T. (2017). Towards targeting PI3K-dependent regulation of gene expression in brain cancer. *Cancers*.

[70] Kusne Y., Carrera-Silva E. A., Perry A. S. (2014). Targeting aPKC disables oncogenic signaling by both the EGFR and the proinflammatory cytokine TNF*α* in glioblastoma. *Science Signaling*.

[71] Ramachandran P., Barria R., Ashley J., Budnik V. (2009). A critical step for postsynaptic F-actin organization: regulation of Baz/Par-3 localization by aPKC and PTEN. *Developmental Neurobiology*.

[72] Manfruelli P., Arquier N., Hanratty W. P., Sémériva M. (1996). The tumor suppressor gene, lethal(2)giant larvae (l(2)gl), is required for cell shape change of epithelial cells during Drosophila development. *Development*.

[73] Gold K. S., Brand A. H. (2014). Optix defines a neuroepithelial compartment in the optic lobe of the Drosophila brain. *Neural Development*.

[74] Halder G., Johnson R. L. (2011). Hippo signaling: growth control and beyond. *Development*.

[75] Reddy B. V. V. G., Rauskolb C., Irvine K. D. (2010). Influence of Fat-Hippo and Notch signaling on the proliferation and differentiation of Drosophila optic neuroepithelia. *Development*.

[76] Kawamori H., Tai M., Sato M., Yasugi T., Tabata T. (2011). Fat/Hippo pathway regulates the progress of neural differentiation signaling in the Drosophila optic lobe. *Development, Growth & Differentiation*.

[77] Grzeschik N. A., Parsons L. M., Allott M. L., Harvey K. F., Richardson H. E. (2010). Lgl, aPKC, and Crumbs Regulate the Salvador/Warts/Hippo Pathway through Two Distinct Mechanisms. *Current Biology*.

[78] Menéndez J., Pérez-Garijo A., Calleja M., Morata G. (2010). A tumor-suppressing mechanism in Drosophila involving cell competition and the Hippo pathway. *Proceedings of the National Acadamy of Sciences of the United States of America*.

[79] Huang J., Wu S., Barrera J., Matthews K., Pan D. (2005). The Hippo signaling pathway coordinately regulates cell proliferation and apoptosis by inactivating Yorkie, the Drosophila homolog of YAP. *Cell*.

[80] Ziosi M., Baena-López L. A., Grifoni D. (2010). dMyc functions downstream of yorkie to promote the supercompetitive behavior of hippo pathway mutant Cells. *PLoS Genetics*.

[81] Neto-Silva R. M., de Beco S., Johnston L. A. (2010). Evidence for a growth-stabilizing regulatory feedback mechanism between Myc and Yorkie, the drosophila homolog of Yap. *Developmental Cell*.

[82] Izergina N., Balmer J., Bello B., Reichert H. (2009). Postembryonic development of transit amplifying neuroblast lineages in the Drosophila brain. *Neural Development*.

[83] Atwood S. X., Prehoda K. E. (2009). aPKC phosphorylates miranda to polarize fate determinants during neuroblast asymmetric cell division. *Current Biology*.

[84] Zhu S., Barshow S., Wildonger J., Jan L. Y., Jan Y.-N. (2011). Ets transcription factor Pointed promotes the generation of intermediate neural progenitors in Drosophila larval brains. *Proceedings of the National Acadamy of Sciences of the United States of America*.

[85] Xie Y., Li X., Deng X. (2016). The Ets protein Pointed prevents both premature differentiation and dedifferentiation of Drosophila intermediate neural progenitors. *Development*.

[86] Downs J. A. (2007). Chromatin structure and DNA double-strand break responses in cancer progression and therapy. *Oncogene*.

[87] Gilbert C. A., Ross A. H. (2009). Cancer stem cells: cell culture, markers, and targets for new therapies. *Journal of Cellular Biochemistry*.

[88] Haenfler J. M., Kuang C., Lee C.-Y. (2012). Cortical aPKC kinase activity distinguishes neural stem cells from progenitor cells by ensuring asymmetric segregation of Numb. *Developmental Biology*.

[89] Bello B., Reichert H., Hirth F. (2006). The brain tumor gene negatively regulates neural progenitor cell proliferation in the larval central brain of Drosophila. *Development*.

[90] Betschinger J., Mechtler K., Knoblich J. A. (2006). Asymmetric segregation of the tumor suppressor brat regulates self-renewal in drosophila neural stem cells. *Cell*.

[91] Eroglu E., Burkard T. R., Jiang Y. (2014). SWI/SNF complex prevents lineage reversion and induces temporal patterning in neural stem cells. *Cell*.

[92] Weng M., Golden K. L., Lee C.-Y. (2010). dFezf/Earmuff Maintains the Restricted Developmental Potential of Intermediate Neural Progenitors in Drosophila. *Developmental Cell*.

[93] Seimiya M., Gehring W. J. (2000). The Drosophila homeobox gene optix is capable of inducing ectopic eyes by an eyeless-independent mechanism. *Development*.

[94] Fults D., Pedone C., Dai C., Holland E. C. (2002). MYC expression promotes the proliferation of neural progenitor cells in culture and in vivo. *Neoplasia*.

[95] Herms J. W., Von Loewenich F. D., Behnke J., Markakis E., Kretzschmar H. A. (1999). C-MYC oncogene family expression in glioblastoma and survival. *World Neurosurgery*.

[96] Faria M. H., Khayat A. S., Burbano R. R., Rabenhorst S. H. (2008). c-MYC amplification and expression in astrocytic tumors. *Acta Neuropathologica*.

[97] Simeone P., Trerotola M., Urbanella A. (2014). A unique four-hub protein cluster associates to glioblastoma progression. *PLoS ONE*.

[98] Chen G., Kong J., Tucker-Burden C. (2014). Human brat ortholog TRIM3 is a tumor suppressor that regulates asymmetric cell division in glioblastoma. *Cancer Research*.

[99] Mongiardi M. P., Savino M., Falchetti M. L. (2016). c-MYC inhibition impairs hypoxia response in glioblastoma multiforme. *Oncotarget *.

[100] Tateishi K., Iafrate A. J., Ho Q. (2016). Myc-Driven glycolysis is a therapeutic target in glioblastoma. *Clinical Cancer Research*.

[101] Galardi S., Savino M., Scagnoli F. (2016). Resetting cancer stem cell regulatory nodes upon MYC inhibition. *EMBO Reports*.

[102] Nolo R., Morrison C. M., Tao C., Zhang X., Halder G. (2006). The bantam MicroRNA is a target of the hippo tumor-suppressor pathway. *Current Biology*.

[103] Weng R., Cohen S. M. (2015). Control of drosophila type I and type II central brain neuroblast proliferation by bantam microRNA. *Development*.

[104] Harvey K. F., Zhang X., Thomas D. M. (2013). The Hippo pathway and human cancer. *Nature Reviews Cancer*.

[105] Liu Y.-C., Wang Y.-Z. (2015). Role of Yes-associated protein 1 in gliomas: pathologic and therapeutic aspects. *Tumor Biology*.

[106] Archibald A., Al-Masri M., Liew-Spilger A., McCaffrey L. (2015). Atypical protein kinase C induces cell transformation by disrupting Hippo/Yap signaling. *Molecular Biology of the Cell (MBoC)*.

[107] Parisi F., Riccardo S., Daniel M. (2011). Drosophila insulin and target of rapamycin (TOR) pathways regulate GSK3 beta activity to control Myc stability and determine Myc expression in vivo. *BMC Biology*.

[108] Mitchell N. C., Tchoubrieva E. B., Chahal A. (2015). S6 Kinase is essential for MYC-dependent rDNA transcription in Drosophila. *Cellular Signalling*.

[109] Zhu J., Blenis J., Yuan J. (2008). Activation of PI3K/Akt and MAPK pathways regulates Myc-mediated transcription by phosphorylating and promoting the degradation of Mad1. *Proceedings of the National Acadamy of Sciences of the United States of America*.

[110] Guo P., Nie Q., Lan J., Ge J., Qiu Y., Mao Q. (2013). C-Myc negatively controls the tumor suppressor PTEN by upregulating miR-26a in glioblastoma multiforme cells. *Biochemical and Biophysical Research Communications*.

[111] McNamara M. G., Sahebjam S., Mason W. P. (2013). Emerging biomarkers in glioblastoma. *Cancers*.

[112] Heddleston J. M., Li Z., McLendon R. E., Hjelmeland A. B., Rich J. N. (2009). The hypoxic microenvironment maintains glioblastoma stem cells and promotes reprogramming towards a cancer stem cell phenotype. *Cell Cycle*.

[113] Duan S., Yuan G., Liu X. (2015). PTEN deficiency reprogrammes human neural stem cells towards a glioblastoma stem cell-like phenotype. *Nature Communications*.

[114] Koul D. (2008). PTEN signaling pathways in glioblastoma. *Cancer Biology & Therapy*.

[115] Tahirovic S., Bradke F. (2009). Neuronal polarity. *Cold Spring Harbor Perspectives in Biology*.

