# Supplementary material for: Failure of the PTEN/aPKC/Lgl Axis Primes Formation of Adult Brain Tumours in Drosophila
Source: Biomed Res Int. 2017 Dec 27;2017:2690187. doi: 10.1155/2017/2690187 (PMC5763105; doi:10.1155/2017/2690187)
Supplement: Supplementary Materials — Supplementary Figure S1. Measurement of the A-P larval brain lobe diameter following neurogenic growth. Supplementary Figure S2. The most part of the immature cells filling the Optix-aPKCCAAX-wt larval brains are type II NBs and INPs. Supplementary Figure S3. The activated form of aPKC induces an increase of the adult fly interocular distance (IOD). Supplementary Figure S4. Kaplan-Meier survival curve of Optix-aPKCCAAX-wt adult flies maintained at 25°C. [file 2690187.f1.pdf]

Failure of the PTEN/aPKC/Lgl Axis Primes Formation of Adult Brain Tumours in *Drosophila*

Simona Paglia, Manuela Sollazzo, Simone Di Giacomo, Dario de Biase, Annalisa Pession and Daniela Grifoni

SUPPLEMENTARY INFORMATION

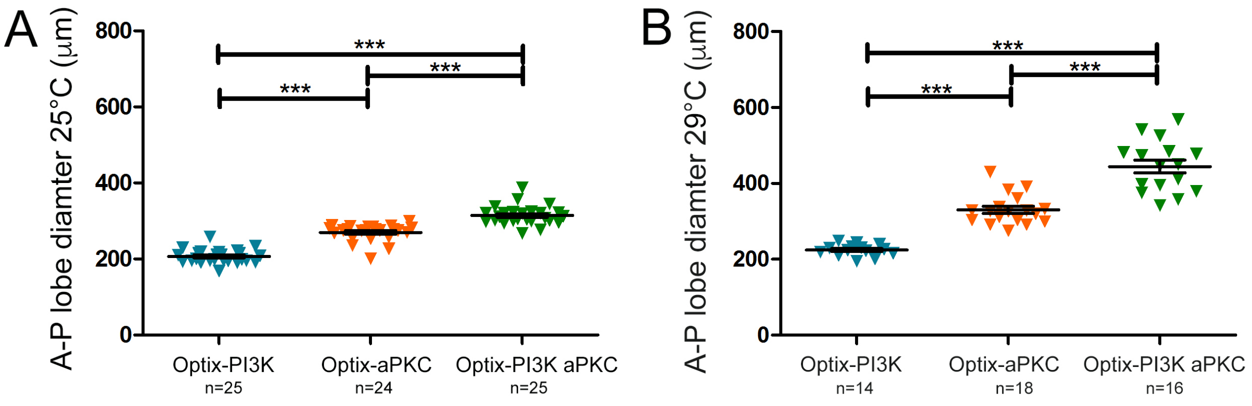

Figure S1. Measurement of the A-P larval brain lobe diameter following neurogenic growth. The graphs display the Anterior-Posterior (A-P) larval brain lobe diameter of the experimental progeny from each group grown at 25°C (A) and at 29°C (B). Each triangle represents one individual, and the black bar indicates the average diameter. n is given for each group. The differences are all statistically significant, $P<0.001$.

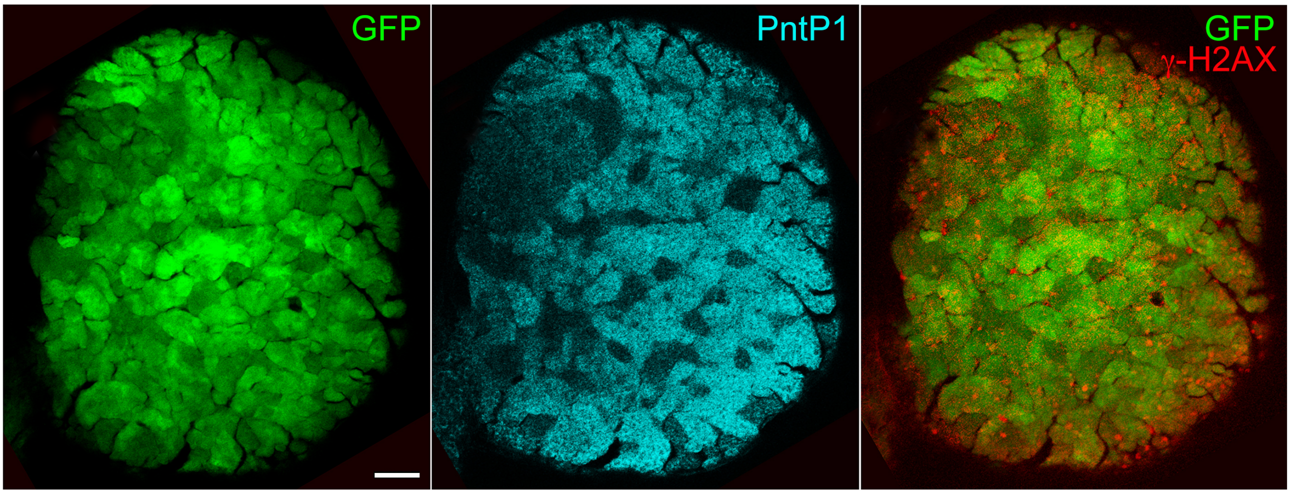

Figure S2. The most part of the immature cells filling the Optix-aPKC$^{CAAX\text{-}wt}$ larval brains are type II NBs and INPs.

Brains from Optix-aPKC$^{CAAX\text{-}wt}$ late L3 larvae grown at 29°C. PntP1 (cyan) stains type II NBs and INPs and γ-H2AX (red) stains DNA double strand breaks. Scale bar is 50μm.

A

	Optix-Gal4					
	PI3K	aPKC	Double	PI3K	aPKC	Double
	25°C			29°C		
Total eclosed adults	455	217	301	173	141	120
Total pharates	5	25	70	11	78	0
Expected experimental progeny	303	108	150	115	93	60
Observed experimental progeny	338	86	77	121	0	0

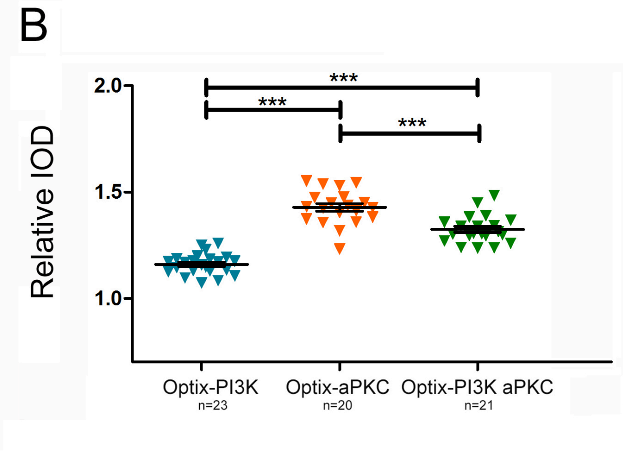

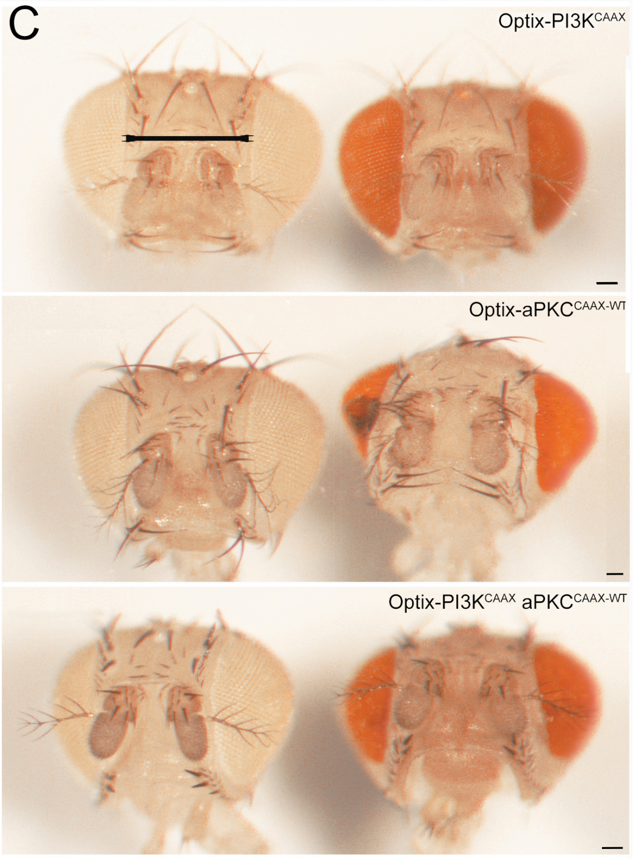

Figure S3. The activated form of aPKC induces an increase of the adult fly interocular distance (IOD).

(A) The table summarises the expected and observed experimental progeny from the activated forms of PI3K and aPKC, separated and combined, under the control of the *Optix* promoter at 25 and 29°C. A X-square test was performed that found no significant differences between the observed (eclosed adults + pharates) and the expected progenies for $P<0.05$, except for the *Optix*-PI3K aPKC progeny at 29°C which resulted completely absent. (B) The graph displays the IOD of the experimental progeny from each group, normalised to the IOD of the respective control progeny. Each triangle represents one individual, and the black bar indicates the average ratio. n is given for each group. The differences are all statistically significant, $P<0.001$. (C) Representative photographs of adult heads from the groups plotted in B. Each transgenic individual (on the right) represents the average phenotype of its class, and is flanked by a sibling representing the average phenotype of its class. The black line indicates the region used for IOD measurement. Scale bars are 50μm.

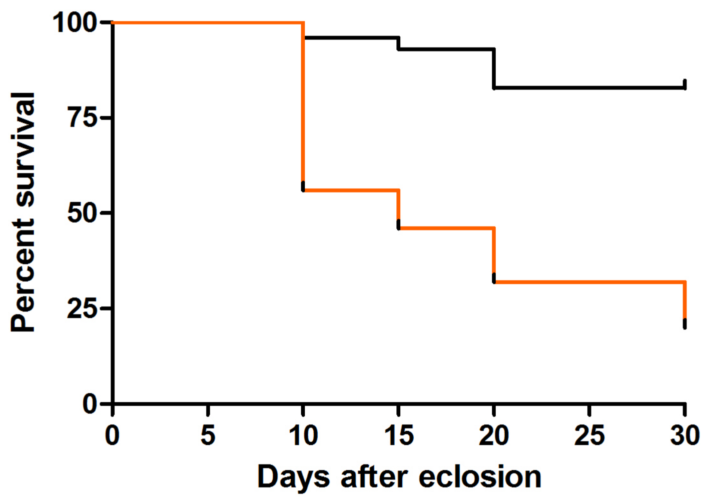

Figure S4. Kaplan-Meier survival curve of Optix-aPKC$^{CAAX\text{-}wt}$ adult flies maintained at 25°C. The graph summarises the survival percentages of Optix-aPKC$^{CAAX\text{-}wt}$ (orange line) and Optix-GFP (black line) sibling flies reared at 25°C. Adult flies were transferred on fresh medium every two days and counted at days 10, 15, 20 and 30 after eclosion.
